# The Value of Electronic Health Records Since the Health Information Technology for Economic and Clinical Health Act: Systematic Review

**DOI:** 10.2196/37283

**Published:** 2022-09-27

**Authors:** Shikha Modi, Sue S Feldman

**Affiliations:** 1 Department of Political Science Auburn University Auburn, AL United States; 2 Department of Health Services Administration University of Alabama at Birmingham Birmingham, AL United States

**Keywords:** electronic health records, EHRs, value, financial outcomes, clinical outcomes, health informatics, clinical informatics

## Abstract

**Background:**

Electronic health records (EHRs) are the electronic records of patient health information created during ≥1 encounter in any health care setting. The Health Information Technology Act of 2009 has been a major driver of the adoption and implementation of EHRs in the United States. Given that the adoption of EHRs is a complex and expensive investment, a return on this investment is expected.

**Objective:**

This literature review aims to focus on how the value of EHRs as an intervention is defined in relation to the elaboration of value into 2 different value outcome categories, financial and clinical outcomes, and to understand how EHRs contribute to these 2 value outcome categories.

**Methods:**

This literature review was conducted using PRISMA (Preferred Reporting Items for Systematic Reviews and Meta-Analyses). The initial search of key terms, EHRs, values, financial outcomes, and clinical outcomes in 3 different databases yielded 971 articles, of which, after removing 410 (42.2%) duplicates, 561 (57.8%) were incorporated in the title and abstract screening. During the title and abstract screening phase, articles were excluded from further review phases if they met any of the following criteria: not relevant to the outcomes of interest, not relevant to EHRs, nonempirical, and non–peer reviewed. After the application of the exclusion criteria, 80 studies remained for a full-text review. After evaluating the full text of the residual 80 studies, 26 (33%) studies were excluded as they did not address the impact of EHR adoption on the outcomes of interest. Furthermore, 4 additional studies were discovered through manual reference searches and were added to the total, resulting in 58 studies for analysis. A qualitative analysis tool, ATLAS.ti. (version 8.2), was used to categorize and code the final 58 studies.

**Results:**

The findings from the literature review indicated a combination of positive and negative impacts of EHRs on financial and clinical outcomes. Of the 58 studies surveyed for this review of the literature, 5 (9%) reported on the intersection of financial and clinical outcomes. To investigate this intersection further, the category “Value–Intersection of Financial and Clinical Outcomes” was generated. Approximately 80% (4/5) of these studies specified a positive association between EHR adoption and financial and clinical outcomes.

**Conclusions:**

This review of the literature reports on the individual and collective value of EHRs from a financial and clinical outcomes perspective. The collective perspective examined the intersection of financial and clinical outcomes, suggesting a reversal of the current understanding of how IT investments could generate improvements in productivity, and prompted a new question to be asked about whether an increase in productivity could potentially lead to more IT investments.

## Introduction

Electronic health records (EHRs) are described as electronic records of patient health information created by ≥1 encounter in any health care setting and include patient demographics, issues, medication information, laboratory data, radiology reports, and history [1]. EHRs enable health information exchange, clinical decision support, diagnostic support, patient health portals, and more [2]. EHR use has the potential to improve the quality of care and patient safety [3] and has become an important part of the modern health system because of government policies, technology developments, health care challenges, and market situations [4]. The Health Information Technology for Economic and Clinical Health (HITECH) Act has been a major driver of the increase in the adoption and implementation of EHRs [5].

The HITECH Act of 2009 was passed to decrease health care costs, improve quality, and increase patient safety through incentives for providers (physicians) and organizations that provided proof of their meaningful use (MU) of certified EHR systems [5]. Approximately US $27 billion in incentives was given to physicians and hospitals that adopted and used EHRs according to federally defined “meaningful use” criteria [6]. Out of US $27 billion, US $406 million was allotted to Medicare Advantage Organizations for eligible providers. The Center for Medicare and Medicaid Services (CMS) provided subsidy payments of US $63,750 over 6 years for Medicaid or US $44,000 over 5 years for Medicare to individual physicians if they used certified EHRs beginning in 2011 and exhibited MU criteria [7]. It is worth noting that in 2018, the CMS refocused MU on increasing health information exchange and patient access to data, renaming MU as Promoting Interoperability Programs.

Given that it has been over a decade since the HITECH Act was passed, sufficient data are available to understand how EHR adoption investment adds value to the hospitals that have EHR systems in place. It is important to first define “value” to understand the value of EHR adoption from a comprehensive perspective.

When reviewing the cost and resources associated with EHR adoption, it is generally considered to be an expensive investment [8,9], with an expectation of a return or value on the investment. Typically, return on investment (ROI) is measured by dividing the net profit by the net investment [10]. ROI-related concerns about EHR adoption were considered to be a major barrier to the adoption of EHRs, primarily as the value was unknown [11]. Jang et al [9] calculated the ROI for EHR adoption by looking at the breakeven point of EHR adoption investment. This study focused on 17 community primary care practices targeting the financial aspect of EHR adoption but did not consider the financial aspect of multilayered decisions such as system selection, employee training, updating or maintaining systems, and training employees for updated systems [11].

Moving beyond ROI, value can be defined as “considering (someone or something) to be important or beneficial” [12]. To simplify this definition, anything that benefits or is important to an individual is considered to be valuable to that individual, regardless of it being an action or intervention. Value is defined in multiple ways within the health care industry. Payne et al [13] describe value as dollars (financial), productivity (clinical), or effectiveness (clinical). Payne et al [13] also suggest that health IT (HIT) literature is primarily focused on productivity (process) and effectiveness (outcome), followed by dollars (outcome). Feldman et al [14] explain value as a combination of tangible (dollars, financial) and intangible (doing the right thing; trust relationships, social) components. In terms of examining the EHR value component, another study analyzed the value of EHRs in terms of efficiency (clinical) and cost savings (financial). This study further used efficiency to derive value by looking at the quality of care and cost savings from better claims management and reduced payments [11]. Riskin et al [15] highlighted the national focus on health reform and defined its value in terms of improved outcomes (clinical) and reduced costs (financial). Yeung [16] discussed EHR in terms of value as it is connected to improving services (clinical) delivered at local health departments. Hepp et al [17] evaluated the value of EHRs by looking at EHRs as a cost-effective strategy to improve medication safety (clinical). Adler-Milstein et al [18] analyzed different scopes of the value of EHRs by gauging process adherence (clinical), patient satisfaction (clinical), and efficiency outcomes (clinical).

The environment in which HIT is used may have an impact on the value that is derived from HIT [19]. For example, Peterson et al [11] suggested that current users of EHR systems focus on value in terms of improving workflows and, as a result, better clinical outcomes, whereas local health departments or community clinics may focus on value in terms of capturing patient information to improve the services that are provided [16] or for ambulatory settings on increasing medication safety [17]. Thinking about EHRs’ value more holistically, the value could equate with increased revenue and reduced cost (financial). For patients, it could mean improved health and prevention of illness (outcomes); for providers, it could signal reduced errors and an increase in the efficiency of care (process); and for the government, it could correspond with improvements in population health through timely public health reporting and population well-being (process and outcomes) [13]

The World Health Organization defines an outcome measure as “a change in the health of an individual, group of people, or population that is attributable to an intervention or series of interventions” [20]. Outcomes, in the conventional health services sense, are usually regarded as clinical outcomes [21]; however, to represent the scope of the Triple Aim of health care, the authors built upon the literature to broaden the definition of outcomes to include financial and social outcomes, in addition to traditional clinical outcomes.

This review of the literature aimed to describe how the value of EHRs, as an intervention, is defined in relation to the elaboration of value into 2 different value outcome categories, financial and clinical outcomes, and by understanding the contributions that EHRs make to these 2 value outcome categories.

## Methods

This review was conducted using the PRISMA (Preferred Reporting Items for Systematic Reviews and Meta-Analyses) [22]. This method has been used for other qualitative analyses of literature and is therefore regarded as a suitable method for this qualitative systematic review of the literature [23,24]. To capture the multidisciplinary evidence in this field, the following databases were used to conduct the initial search: PubMed, Scopus, and Embase. To capture the decade that followed the enactment of the HITECH Act, the literature published in English between January 2009 and December 2019 was used as a filter to refine the results. The initial keywords used were “electronic health records,” “EHR,” “value,” “financial outcomes,” and “clinical outcomes.” To ensure the comprehensiveness of the literature search, all the outcome categories were searched separately and in conjunction with one another. The search strings and gathered results were extensive and lengthy and are recorded in Table 1. To optimize the chance of finding relevant studies on the value of EHR from the financial and clinical outcomes perspective after the enactment of the HITECH Act, the following filters were applied to the searches: (1) keywords in the title or abstract, (2) published in English, (3) published in the United States only, and (4) published between 2009 and 2019, when applicable.

**Figure 1:**
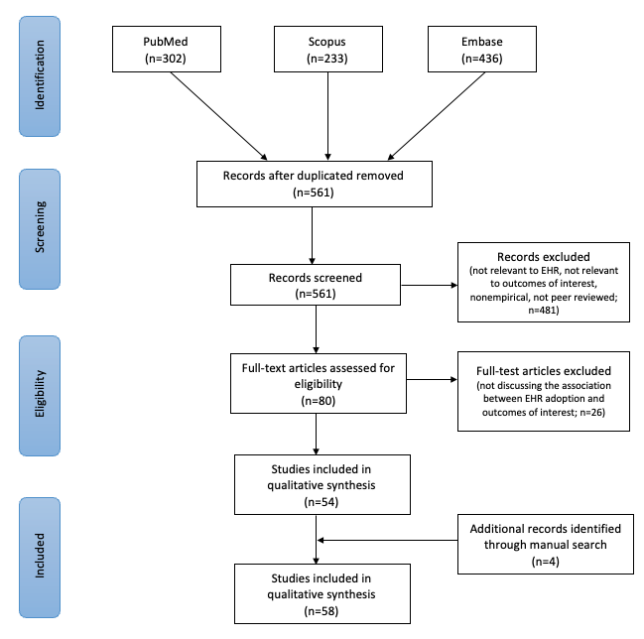
PRISMA (Preferred Reporting Items for Systematic Reviews and Meta-Analyses) flow diagram [22]. EHR: electronic health record.

A total of 971 articles was included in the initial literature screening, of which, after removing 410 (42.2%) duplicates, 561 (57.8) were incorporated in the title and abstract screening. During the title and abstract screening phase, articles were excluded from further review phase if they met any of the following criteria: (1) not relevant to the outcomes of interest, (2) not relevant to EHRs, (3) nonempirical, and (4) non–peer reviewed. After the application of the exclusion criteria, 80 studies remained for a full-text review. After evaluating the full text of the residual 80 studies, 26 (33%) studies were excluded as they did not address the impact of EHR adoption on the outcomes of interest. Following this, 4 additional studies were discovered through manual reference searches and were added to the total, resulting in 58 studies for analysis. Figure 1 displays this process in a flow diagram. Both authors were involved in the article search, selection, and review process.

The 58 studies selected for inclusion are exhibited in the *Results* section and are organized by outcome category. ATLAS.ti (version 8.2), a qualitative data analysis tool, was used to categorize and code the final 58 studies. All studies were uploaded into ATLAS.ti as full-text documents with names that included the first author, year of publication, and article title. Qualitative data analysis software was deemed fitting for this type of analysis as it allows for the possibility of applying a recurring and reiterative approach to data analysis that is efficient and would have been difficult to replicate using a spreadsheet application [25].

The coding process began by analyzing each article to understand the context in relation to how each outcome category is defined in the literature and learn about the evaluation process of the impact of EHRs on these outcome categories. For this study, overarching a priori categories (financial outcomes and clinical outcomes) were used, and the studies were further categorized under these 2 overarching categories. Additional categories that were developed included the following:

- Financial outcomes: cost, revenue, profit margins, reimbursement, and return on assets
- Clinical outcomes: productivity, workflow efficiency, medical errors, patient safety, patient satisfaction, clinical volume, readmission rates, length of stay (LOS), and quality indicators at individual patient levels

Additional categories were added as necessitated throughout the coding and category generation process, which was part of the larger data analysis process. For example, introduction and gap categories were generated as they assisted in the writing of the introduction and gap and supplied context for this review of literature; however, quotations included in these categories did not necessarily factor into the results presented.

**Table 1:** Search strings from the literature search for the impact of electronic health records on financial and clinical outcomes (N=971).

Database and keywords			Results, n (%)		Filters		Results after applying filters, n (%)
**PubMed**							
	([([([(((Finance*[Title] OR monetary[Title] OR economic*[Title] OR fiscal[Title] OR commercial[Title] OR cost[Title])) OR (Finance*[Other Term] OR monetary[Other Term] OR economic*[Other Term] OR fiscal[Other Term] OR cost[Other Term])) OR “Economics” [Mesh]]) OR ([(Clinical[Title] OR quality[Title] OR)] OR [Clinical[Other Term] OR quality[Other Term]]) AND ((((((Adopt*[Title] OR (Adopt*[Other Term]) OR implement*(Title)] OR implement*[Other Term])] AND [([(Follow-up-stud*[Title] OR prognos*[Title] OR predict*[Title] OR course[Title] OR followup-stud*[Title] OR efficacy[Title] OR complication[Title] OR chang*[Title] OR effective*[Title] OR evaluat*[Title] OR improve*[Title] OR indicat*[Title] OR impact*[Title] OR consequence*[Title] OR development*[Title] OR Result*[Title] OR outcome*[Title])] OR [Follow-up-stud*(Other Term) OR prognos*[Other Term] OR predict*(Other Term) OR course(Other Term) OR followup-stud*(Other Term) OR efficacy(Other Term) OR complication(Other Term) OR chang*(Other Term) OR effective*(Other Term) OR evaluat*(Other Term) OR improve*(Other Term) OR indicat*(Other Term) OR impact*(Other Term) OR consequence*(Other Term) OR development*(Other Term) OR Result*(Other Term) OR outcome*(Other Term)]) OR “follow-up studies” (mesh)]) AND ([([Electronic-health-record*(Title) OR electronic-medical-record*(Title) OR computerized-health-record*(Title) OR computerized-medical-record*(Title) OR EHR(Title) OR electronic-patient-record*(Title)]) OR (Electronic-health-record*[Other Term] OR electronic-medical-record*[Other Term] OR computerized-health-record*[Other Term] OR computerized-medical-record*[Other Term] OR EHR[Other Term] OR electronic-patient-record*[Other Term])] OR “electronic health records” [mesh])	193 (19.9)		Years: 2009-2019; language: English		179 (18.4)	
	([“electronic health records adoption”(Title/Abstract)] OR “EHR adoption”[Title/Abstract]) AND “financial outcomes”(Title/Abstract)	0 (0)		N/A^a^		0 (0)	
	([“electronic health records adoption”(Title/Abstract)] OR “EHR adoption”[Title/Abstract]) AND “financial”(Title/Abstract)	39 (4)		Years: 2009-2019; language: English		33 (3.4)	
	([“electronic health records adoption”(Title/Abstract)] OR “EHR adoption”[Title/Abstract]) AND “clinical outcomes”(Title/Abstract)	1 (0.1)		Years: 2009-2019; language: English		1 (0.1)	
	([“electronic health records adoption”(Title/Abstract)] OR “EHR adoption”[Title/Abstract]) AND “clinical”(Title/Abstract)	99 (10.2)		Years: 2009-2019; language: English		89 (9.2)	
**Scopus**							
	(TITLE-ABS-KEY [electronic-health-record* OR electronic-medical-record* OR computerized-health-record* OR computerized-medical-record* OR ehr OR electronic-patient-record* OR “electronic health record”] AND TITLE-ABS-KEY [finance* OR monetary OR economic* OR fiscal OR “economic”] AND TITLE-ABS-KEY [clinical OR quality] AND TITLE-ABS-KEY [“follow-cup studies” OR follow-up-stud* OR prognos* OR predict* chang* OR effective* OR evaluat* OR improve* OR indicat* OR impact* OR consequence* OR outcome*] AND TITLE-ABS-KEY [Adopt* OR implement*])	70 (7.2)		Years: 2009-2019; language: English; country: United States		35 (3.6)	
	TITLE-ABS-KEY (“EHR adoption” OR “electronic health records adoption” AND “financial outcomes”)	0 (0)		N/A		0 (0)	
	TITLE-ABS-KEY (“EHR adoption” OR “electronic health records adoption” AND “financial”)	61 (6.3)		Years: 2009-2019; language: English; country: United States		41 (4.2)	
	TITLE-ABS-KEY (“ehr adoption” OR “electronic health records adoption” AND “clinical outcomes”)	2 (0.2)		Years: 2009-2019; language: English; country: United States		2 (0.2)	
	TITLE-ABS-KEY (“EHR adoption” OR “electronic health records adoption” AND “clinical”)	173 (17.8)		Years: 2009-2019; language: English; country: United States		155 (16)	
**Embase**							
	(“electronic health record*”:ti,ab,kw OR “electronic medical record*”:ti,ab,kw OR “computerized health record*”:ti,ab,kw OR “computerized medical record*”:ti,ab,kw OR ehr:ti,ab,kw OR “electronic patient record*”:ti,ab,kw OR “electronic health record”:ti,ab,kw) AND (finance*:ti,ab,kw OR monetary:ti,ab,kw OR economic*:ti,ab,kw OR fiscal:ti,ab,kw OR “economic”:ti,ab,kw) AND (clinical:ti,ab,kw OR quality:ti,ab,kw) AND (“follow-up studies”:ti,ab,kw OR “follow up stud*”:ti,ab,kw OR prognos*:ti,ab,kw OR predict*:ti,ab,kw OR course:ti,ab,kw OR “followup stud*”:ti,ab,kw OR efficacy:ti,ab,kw OR complication:ti,ab,kw OR chang*:ti,ab,kw OR effective*:ti,ab,kw OR evaluat*:ti,ab,kw OR imptove*:ti,ab,kw OR indicat*:ti,ab,kw OR impact*:ti,ab,kw OR consequence*:ti,ab,kw OR development*:ti,ab,kw OR result*:ti,ab,kw OR outcome*:ti,ab,kw) AND (adopt*:ti,ab,kw OR implement*:ti,ab,kw)	350 (36)		Years: 2009-2019		303 (31.2)	
	(“electronic health records adoption”:ti,ab,kw OR “ehr adoption”:ti,ab,kw) AND “financial outcomes”:ti,ab,kw	0 (0)		N/A		0 (0)	
	(“electronic health records adoption”:ti,ab,kw OR “ehr adoption”:ti,ab,kw) AND “financial”:ti,ab,kw	42 (4.3)		Years: 2009-2019		35 (3.6)	
	(“electronic health records adoption”:ti,ab,kw OR “ehr adoption”:ti,ab,kw) AND “clinical outcomes”:ti,ab,kw	3 (0.3)		Years: 2009-2019		3 (0.3)	
	(“electronic health records adoption”:ti,ab,kw OR “ehr adoption”:ti,ab,kw) AND “clinical”:ti,ab,kw	104 (10.7)		Years: 2009-2019		95 (9.8)	

^a^N/A: not applicable.

## Results

Information from the reviewed articles (n=58) was analyzed to ascertain how the value of EHRs is determined regarding financial and clinical outcomes relative to how they are defined earlier in this paper. In addition, findings from this review of the literature describe how EHR adoption affects each outcome category.

### Financial Outcomes

Of the 58 studies reviewed, 21 (36%) studies incorporated segments that were coded under the “Value-Financial Outcomes” category. Different measures of financial outcomes were used in these studies, such as cost [26-29], revenue [28,29], profit margins [8,27], reimbursement [30], and return on assets [8]. These different financial outcome measures are described and detailed in Table 2. The included studies contained positive (17/58, 81%), negative (4/58, 19%), and no (3/58, 14%) association relationships between EHR adoption and financial outcomes. There were overlapping positive and negative impacts of EHR adoption on financial outcomes in some of the reviewed studies.

**Table 2:** Reviewed studies on the impact of EHR^a^ adoption and financial and clinical outcomes.

Study	Journal or conference	Study period or data set	Objective	Outcome measures	Financial (n=21)	Clinical (n=54)
Adler-Milstein et al [18]	*Health Services Research*	AHA IT Supplement Survey (2008-2011), AHA Annual Survey (2009-2012), CMS^b^ Hospital Compare data set (2009-2012), and CMS EHR Incentive Program Reports	To examine the relationship between EHR adoption and hospital outcomes	Efficiency (measured by the ratio of a hospital’s total expenditures to adjusted patient days), process adherence, and patient satisfaction	✓^c^	✓
Appari et al [31]	*The American Journal of Managed Care*	Cross-sectional retrospective study, data on hospital patient safety performance (2008-2010) combined with IT systems data (2007; n=3002 nonfederal acute care hospitals)	To determine whether HIT^d^ systems are associated with better patient safety in acute care settings	Adverse event indicators developed by AHRQ^e^ (death among surgical patients with serious, treatable complications; collapsed lung that results from medical treatment [iatrogenic pneumothorax]; breathing failure after surgery [postoperative respiratory failure]; blood clots in the lung or a large vein after surgery [postoperative pulmonary embolism or deep venous thrombosis]; wounds that split open after surgery [postoperative wound dehiscence]; accidental cuts and tears [accidental puncture or laceration]; death after surgery to repair a weakness in the abdominal aorta [abdominal aortic aneurysm mortality rate]; and death among patients with hip fractures [hip fracture mortality rate])		✓
Bae et al [32]	*BioMed Central Health Services Research*	National Ambulatory Medical Care Survey (37,962 patient visits to 1470 primary care physicians from 2006 to 2009)	To analyze the impact of EHRs on primary care physicians’ workloads	Duration measured in minutes of the face-to-face encounter between physicians and patients (patient face time) for direct patient care during the office visit and number of total patient office visits per physician per week (patient volume)		✓
Behkami et al [33]	*Studies in Health Technology and Informatics*	Simulation of clinic-type scenarios to capture the dynamic nature of policy interventions that affect the adoption of EHR	To describe a framework that allows decision-makers to efficiently evaluate factors that affect EHR adoption and test financial incentives	Revenue	✓	
Bishop et al [34]	*Health Affairs*	Interviews of medical group leaders (n=21) who use electronic communication with patients extensively and staff from 6 of the groups	To understand how primary care practices can use electronic communication to manage clinical issues that are usually managed during clinic visits; determine perceived advantages and disadvantages of the electronic communication programs for patients, physicians, and practices; and determine the barriers to and facilitators of the implementation of the electronic communication programs	The convenience of access, patient satisfaction, efficiency, safety and quality of care, and workload		✓
Brown Jr et al [29]	*Journal of Addiction Medicine*	Data collected from paper patient charts (for preimplementation data) and electronic patient charts (for postimplementation data); patients, clinicians, and management stakeholders participated in surveys	To evaluate the impact of an EMR^f^ system on the Opioid Agonist Treatment Program	Financial performance (revenue), quality (timeliness of medical assessments), productivity (clinic visits), patient satisfaction, and risk management (incident reports)	✓	✓
Bucher et al [35]	*Journal of the American College of Surgeons*	CMS SCIP^g^ measuring compliance rates; HIMSS^h^ hospital EHR adoption survey from 2006 to 2012	To analyze the impact of EHR adoption on hospital compliance with quality and process measures	Hospital compliance with SCIP core measures		✓
Burke et al [36]	*Journal of Innovation in Health Informatics*	Notes of outpatients with type 2 diabetes analyzed (n=537) for 5.5 years	To analyze the impact of EHR use on clinical quality measures and HbA_1c_^i^	HbA_1c_ values		✓
Cheriff et al [37]	*International Journal of Medical Informatics*	The practice management system used to extract physician productivity data (n=203)	To describe the changes in physician productivity in an academic multispecialty group because of ambulatory EHR adoption	Average monthly charge, visit volume, and work-relative value units	✓	✓
Chiang et al [38]	*Journal of American Association for Pediatric Ophthalmology and Strabismus*	Academic pediatric ophthalmology practice data for the year 2006 (n=4 faculty providers)	To analyze the impact of EHR implementation on the volume and time for pediatric ophthalmology	Clinical volume		✓
Chiang et al [39]	*Transactions of the American Ophthalmological Society*	Outpatient clinical examinations (n=120,490) from faculty providers (n=23) at an academic ophthalmology department analyzed for 3 years	To evaluate clinical volume, time requirements, and nature of clinical documentation related to EHR implementation	Clinical volume, time requirements, and nature of clinical documentation		✓
Choi et al [40]	*Journal of Medical Systems*	Retrospective chart review study—a convenience sample of 60 to 80 charts reviewed every month from (January 1, 2006, to October 4, 2009, n=3997; October 5, 2009, to December 31, 2010, n=984)	To analyze the organizational performance and regulatory compliance before and after implementation of the Anesthesia Information Management System	Documentation of medication and patient status		✓
Collum et al [8]	*Healthcare Management Review*	AHA^j^ Annual survey (2007-2010), AHA IT Supplement (2007-2010), and CMS Medicare Cost Reports (2007-2011)	To examine how EHR adoption affects hospital financial performance	Profit margins and return on assets	✓	
Dandu et al [41]	*Clinical Orthopedics and Related Research*	Data were collected from a combination of the Physician Compare data set (2016), Meaningful Use Eligible Professional public use files (2011-2016), and Medicare Utilization and Payment data sets (2012-2016)	To evaluate the impact of EHRs on provider productivity, billing, and orthopedic surgery	Billing, outpatient volume, and surgical volume	✓	✓
Daniel et al [42]	*Academic Emergency Medicine*	Health plan and electronic hospital data from a large urban ED^k^ (November 1, 2004, to March 31, 2005, n=1509 ED encounters compared with September 1, 2005, to February 17, 2006, n=779 ED encounters)	To evaluate the use of paper-based EHR in an ED on LOS^l^ and plan payments	Plan payment for ED encounters and ED LOS	✓	✓
Deily et al [43]	*Health Research and Educational Trust*	Administrative claims data in Pennsylvania from 1998 to 2004 (n=491,832)	To examine whether HIT at nonhospital facilities improves health outcomes and decreases resource use at hospitals within the same network and whether the effect of HIT differs as providers obtain more experience with it	Incidence of obstetric trauma and preventable complications; LOS		✓
Edwardson et al [44]	*Medical Care Research and Review*	Financial panel data from the pediatric primary care network comprising 260 providers across 42 practices (2008-2013)	To examine the effect of EHR adoption on charge capture	Average per-patient charge, average per-patient collections, and charge-to-collection ratios	✓	
Ehrlich et al [45]	*Applied Clinical Informatics*	Survey responses from 32 ophthalmologists after implementation, 28 at 3 months, 35 at 7 months, 40 at 13 months, and 39 at 24 months after implementation (implementation in 2012)	To comprehend and describe the perceptions of ophthalmologists during EHR implementation in an academic department of ophthalmology	Documentation quality, workflow, and efficiency		✓
Flatow et al [46]	*Applied Clinical Informatics*	Retrospective chart review for all patients admitted to the surgical intensive care unit (n=3742; January 1, 2009, to December 31, 2010)	To evaluate key quality measures of a surgical intensive care unit following EHR implementation in a tertiary hospital	LOS, mortality, central line–associated bloodstream infection rates, clostridium difficile colitis rates, readmission rates, and number of coded diagnoses		✓
Furukawa et al [47]	*Journal of the American Medical Informatics Association*	Data collected from Medicare Patient Safety Monitoring System (2010-2013) and HIMSS Analytics database (2008-2013)	To evaluate the impact of meaningful use capabilities on in-hospital adverse drug events	Rate of adverse drug events		✓
Han et al [48]	*American Journal of the Medical Sciences*	A prospective observational study (n=797 patients) at an urban teaching hospital from July 2010 to June 2011 in the MICU^m^	To determine the effect of EHR on MICU mortality, hospital LOS, and medication errors	MICU mortality, hospital LOS, and medication errors		✓
Hepp et al [17]	*Value in Health*	The decision-analytic model was used to estimate the cost-effectiveness of CPOE^n^ in a multidisciplinary medical group for the years 2010 to 2014 (n=400 providers)	To assess the cost-effectiveness of CPOE in the reduction of medication errors and adverse drug events in an ambulatory setting	Costs (CPOE system costs, personnel costs, administrative costs, and prescribing costs), financial incentives (Health Information Technology for Economic and Clinical Health meaningful use incentives and pay-for-performance incentives), medication error probability, and adverse drug event probability	✓	✓
Herasevich et al [49]	*Critical Care Medicine*	A prospective study at Mayo Clinic, Rochester, Minnesota (n=1159 patients) from February 16, 2008, to February 16, 2009	To design and test an electronic algorithm that includes patient characteristics and ventilator settings, allowing notification to bedside providers about potentially injurious ventilator settings to improve the safety of ventilator care and decrease the risk of ventilator-related lung injury	Prevalence of acute lung injury		✓
Hessels et al [50]	*Online Journal of Nursing Informatics*	Data on 854,258 adult patients discharged from 70 New Jersey hospitals and 7679 nurses working in those same hospitals for the year 2006	To examine the relationship between the EHR adoption stage, missed nursing care, nursing practice environment, and adverse outcomes and satisfaction of patients who are hospitalized	Prolonged LOS and patient satisfaction		✓
Howley et al [51]	*Journal of the American Medical Informatics Association*	Compared practice productivity and reimbursement of ambulatory practices (n=30) for 2 years after EHR implementation to their per-EHR implementation baseline	To evaluate how EHR implementation affects the financial performance of ambulatory practices	Reimbursement and practice productivity (number of patient visits)	✓	✓
Jones et al [52]	*American Journal of Managed Care*	Database with 2021 hospitals collected by linking the AHA Annual Survey database, Hospital Compare database, and HIMSS database for the years 2004 and 2007	To analyze longitudinal data on EHR adoption to evaluate the impact of new EHR adoption on quality improvement	Composite measures of hospital process quality for acute myocardial infarction, health failure, and pneumonia		✓
Katzer et al [53]	*Applied Clinical Informatics*	Prehospital patient care reports (n=154) at Georgetown University’s student-run Emergency Medical Services organization	To describe whether implementing an electronic patient care report system influenced improvement in physical exam documentation	Mean physical exam documentation		✓
Kritz et al [54]	*Journal of Evaluation in Clinical Practice*	Opioid treatment program clinics (7 clinics) in New York State—paper patient charts and electronic patient charts (to analyze pre- and postimplementation data), assessment meetings and surveys with patients, direct care providers, and supervisors or managers	Prospective, comparative study using a pre- and postimplementation design to establish whether EHR implementation yielded any improvements	Revenue, quality, productivity, risk management, and satisfaction	✓	✓
Lam et al [55]	*BioMed Central Health Services Research*	Data from physicians with practices at the University of Washington Department of Ophthalmology for the years 2008 to 2012 (n=8 physicians)	To analyze the impact of EHR adoption on patient visit volume at an academic ophthalmology department	Patient volume per provider		✓
Lim et al [28]	*Journal of American Medical Association Ophthalmology*	Population-based, cross-sectional study (n=348)	To evaluate the adoption rate and perceptions of financial and clinical outcomes of EHRs among ophthalmologists in the United States	Net revenues and productivity	✓	✓
Love et al [3]	*Journal of American Medical Informatics Association*	2007 state-wide survey of Massachusetts physicians (n=541)	To characterize and describe physicians’ attitudes toward EHR’s potential to cause new errors, improve health care quality, and change physician satisfaction	Medical errors, quality of care, and physician satisfaction		✓
Lowe et al [56]	*Journal of Wound Ostomy Continence Nurses Society*	Data were collected from a regional Veterans Affairs database and computerized patient medical records for a year after implementation of the EMR wound care template (October 1, 2006, to September 30, 2007) and 2 years before the intervention	To evaluate the impact of a 1-year intervention of an EMR wound care template on the completeness of wound care documentation and medical coding and compare results with the preintervention period	Documentation of wound care and documentation of coding for diagnoses and procedures		✓
McCullough et al [57]	*Health Affairs*	AHA Annual Survey, HIMSS Analytics, and CMS Hospital Compare database for the years 2004 to 2007 (n=3401 nonfederal acute care US hospitals)	To analyze the impact of HIT on the quality of care in US hospitals	Quality indicators: percentage of patients with heart failure given angiotensin-converting enzyme inhibitor or angiotensin II receptor blocker for left ventricular systolic dysfunction; the percentage of smokers with heart failure and pneumonia who were given smoking cessation advice; the percentage of patients with pneumonia assessed and given pneumococcal vaccination if indicated; the percentage of patients with pneumonia whose initial blood culture in the ED preceded their first dose of the hospital-administered antibiotics; and the percentage of patients with pneumonia given the most appropriate initial antibiotic		✓
McCullough et al [58]	*Generating Evidence and Methods to improve patient outcomes)*	Manual review of the paper and electronic charts for 6007 patients across 35 small primary care practices	To analyze the quality measure performance in small practices before and after EHR adoption	Clinical quality measures: antithrombotic therapy, BMI recorded, smoking status recorded, smoking cessation intervention offered, HbA_1c_ testing and control, cholesterol testing and control, and BP^o^ control		✓
Mirani and Harpalani [27]	*ACM Transactions on Management Information Systems*	“Data and Reports” and “Hospital Cost Report” from the CMS website for 2008 to 2010	To analyze the impact of the Medicare EHR incentive program on acute care hospitals	The average cost of ancillary services per patient, profit margins, inpatient bed debts, outpatient bed debts, and patient stay durations	✓	✓
Mitchell et al [59]	*The Journal of Rural Health*	AHA EHR adoption survey and CMS Hospital Compare data set for the year 2009	To investigate whether there is an association between clinical decision support system use and quality disparities in pneumonia process indicators between rural and urban hospitals	Percentage of hospitals meeting quality requirements and pneumonia process composite scores		✓
Patterson et al [60]	*Applied Clinical Informatics*	Data used from the AHA Health IT survey and Medicare Part A claims (n=52,048 Medicare beneficiaries discharged for heart failure anytime during the calendar year 2008)	To compare 30 days all-cause readmission rates for Medicare patients with health failure discharged from hospitals with fully implemented comprehensive EHR vs without it	30-day all-cause readmission rates		✓
Persell et al [61]	*Medical Care*	Time series analysis at a large internal medicine practice from February 1, 2007, to February 1, 2009 (n=12,299 patients eligible at the beginning of the intervention)	To implement and analyze a multifaceted quality improvement intervention using EHRs as tools for improving performance	Quality measures pertaining to coronary heart disease, health failure, diabetes mellitus, and prevention		✓
Radley et al [62]	*Journal of American Medical Informatics Association*	Systematic literature review and random-effects meta-analytic techniques, American Society of Health System Pharmacists Annual survey (2007), AHA Annual Survey (2007), and AHA EHR Adoption Database supplement (2008)	To analyze the adoption of CPOE systems on the reduction in medication errors in hospitals	Likelihood of medication errors		✓
Rao et al [63]	*Journal of American Medical Informatics Association*	Mailed surveys to a nationally representative random sample of practicing physicians from the Physician Masterfile of the American Medical Association (n=2769)	To analyze variation in the adoption of EHR functionalities and their use patterns, barriers to adoption, and perceived benefits by physician practice size	Physician perceptions of quality of clinical decision, quality of communication with patients and other providers, delivery of preventive or chronic care that met the guidelines, avoiding medication errors and prescription refills		✓
Risko et al [64]	*Healthcare*	Patient processing metrics (n=374 observations) were collected for ED physicians (34 physicians) at 2 hospitals for 7 months before and 10 months after EHR implementation	To analyze the impact of EHR implementation on ED physician efficiency and patient throughput	Patient workup times and LOS		✓
Ryan et al [65]	*Medical Care*	Data collected from 143 practices with EHR implementation (2009-2011)	To analyze whether EHR implementation and complementary interventions, such as clinical decision support, technical assistance, and financial incentives improved, the quality of care provided	Quality of care was analyzed from 8 separate indicators; 4 cardiovascular measures (smoking cessation intervention, BP control, cholesterol control, and aspirin or antithrombotic treatment) and 4 additional clinically important measures (BMI measurement, HbA_1c_ control, pneumococcal vaccine, and asthma control)		✓
Schreiber and Shaha [66]	*Journal of Innovation in Health Informatics*	Data collected from a community hospital for 5 years after CPOE adoption	To evaluate whether an increase in adoption of CPOE leads to a decrease in LOS	LOS and cost measured by LOS	✓	✓
Scott et al [67]	*The Journal of Bone and Joint Surgery*	Data collected from an outpatient adult reconstruction clinic (n=143 patients) before implementing the hospital system–wide EMR system and 2 months, 6 months, and 2 years after implementation	To evaluate the impact of EMR implementation using advanced cost-accounting methods on orthopedic surgeons in an outpatient setting	Labor cost, documentation time for providers, and time spent interacting with patients	✓	✓
Shen et al [68]	*International Journal of Healthcare Technology and Management*	National Inpatient Sample and AHA EHR implementation survey for the year 2009	To examine how EHR adoption affected the cost of care and quality outcomes in an acute care hospital setting	Cost of care for the 8 quality indicators (cardiovascular and cerebrovascular) and quality indicators for 5 cardiovascular and 3 cerebrovascular conditions and procedures	✓	✓
Silow-Carroll et al [69]	*Issue Brief (Commonwealth Fund)*	Interviews with individuals in the 9 hospitals that implemented a comprehensive EHR system	To analyze the experience of 9 hospitals in using EHR to improve quality and efficiency	Communication among providers, care coordination, patient engagement, and medical errors		✓
Singh et al [70]	*Journal of American Medical Association Ophthalmology*	Retrospective case-control study comparing the pre- (n=13,969 patient encounters) and post-EHR (n=14,191 patient encounters) implementation periods at an eye institute	To evaluate the impact of EHR system implementation from clinical and economic perspectives at a large multidisciplinary ophthalmic practice	Net revenue, revenue to volume ratio, capital and implementation costs, EHR incentive payments received, patient volume, diagnostic and procedure volume, and coding volumes	✓	✓
Sockolow et al [30]	*Applied Clinical Informatics*	Pre- and postobservational mixed methods study, Philadelphia-based homecare agency with 137 clinicians—data included clinician EHR documentation completion, EHR use data, Medicare billing data, an EHR Nurse Satisfaction survey, clinician observations, clinician interviews, and patient outcomes	To compare workflows, financial billing, and patient outcomes before and after implementation to analyze the effect of a homecare point of care EHR	Number of days required to create a financial reimbursement bill, productivity, behavioral outcomes, and clinicians’ perceptions of patient safety	✓	✓
Thirukumaran et al [71]	*Health Services Research*	Data collected from the SCIP Core Measure data set from the CMS Hospital Inpatient Quality Reporting (n=1816) program (March 2010 to February 2012)	To evaluate the effect of EHR placement on SCIP measures in a tertiary care teaching hospital	SCIP scores		✓
Tidwell et al [72]	*Obstetrics and Gynecology*	Data collected from an obstetrics and gynecology practice comprising 6 physicians and 6 midwives with 150 daily visits	To evaluate whether a low-cost electronic practice management system (EHR) can improve care coordination and financial measures	Net profit, days in accounts receivables, patient visits, no-show rate, and quality data gathering	✓	✓
Varpio et al [73]	*Medical Education*	A 2-phase longitudinal study; data collected through field observations (146 hours with 300 providers, 22 patients, and 32 patient family members), think-aloud (n=13) and think-after (n=11) sessions, interviews (n=39) and document retrieval (n=392)	To evaluate the impact of adopting EHR on clinician experience	Clinician experience was measured in terms of cognitive workload, clinical reasoning support mechanisms, and knowledge about the patient		✓
Walker-Czyz et al [74]	*Journal of Nursing Administration*	Data for a quantitative, retrospective analysis collected from urban hospitals (431 beds) with 10 medical-surgical units and 2 critical care units	To evaluate how an integrated EHR innovation adoption affects cost, nurse satisfaction, and nursing care delivered in terms of quality	Cost (nurse hours per patient day, nurse turnover, and nurse overtime), quality nursing care outcomes (hospital-acquired falls and pressure ulcers, ventilator-associated pneumonia, central line–associated bloodstream infections, and catheter-associated urinary tract infections)	✓	✓
Wang et al [75]	*Preventing Chronic Disease*	Clinical quality measure performance data collected from 151 primary care practices that implemented EHR (October 2009 to October 2011)	To analyze how clinical quality measures for independent primary care practices improve as a result of EHR use and technical support from a local public health agency	4 key quality measures: antithrombotic therapy, BP control, HbA_1c_ testing, and smoking cessation intervention		✓
Wang et al [26]	*International Journal of Accounting Information Systems*	Definitive health care data set for hospital-level data for the years 2011 to 2016 (n=3266 observations)	To evaluate how HIT expenses and intermediate business processes affect hospital financial performance and productivity	Return on assets, productivity ([net revenue, 1 million]), and number of staff beds)	✓	✓
Xiao et al [76]	*Perspectives in Health Information Management*	Charts were reviewed to collect data from a large tertiary public medical center (3 years before and 3 years after EHR implementation in July 2009)	To describe how electronic charting implementation in a large public outpatient clinic improves clinical documentation	Note completion and documentation of medication		✓
Yeung [16]	*International Journal of Medical Informatics*	433 local health departments’ population-based data for 433 counties	To determine the impact of the adoption of EHR and health information exchange changes by local health departments on population health	The health of a population at the county level, as measured by health outcomes such as premature death and health-related quality of life		✓
Wani and Malhotra [77]	*Journal of Operations Management*	Acute care hospitals in California	To analyze the impact of EHR adoption in terms of full adoption vs meaningful assimilation on clinical outcomes	LOS and readmission rates		✓
Zhou et al [78]	*Journal of the American Medical Informatics Association*	To evaluate the extent of EHR use and how the quality of care delivered in ambulatory care practices varied according to the duration of EHR availability	Quality measures are aggregated into 6 clinical categories (asthma care, behavioral and mental health, cancer screening, diabetes care, well-child and adolescent visits, and women’s health screenings)	Quality measures aggregated into 6 clinical categories (asthma care, behavioral and mental health, cancer screening, diabetes care, well child and adolescent visit, women’s health screenings)	✓	

^a^EHR: electronic health record.

^b^CMS: Centers for Medicare and Medicaid Services.

^c^✓: indicates that the outcome was discussed in the study.

^d^HIT: health IT.

^e^AHRQ: Agency for Healthcare Research and Quality.

^f^EMR: electronic medical record.

^g^SCIP: Surgical Care Improvement Project.

^h^HIMSS: Healthcare Information and Management Systems Society.

^i^HbA_1c_: hemoglobin A_1c_.

^j^AHA: American Hospital Association.

^k^ED: emergency department.

^l^LOS: length of stay.

^m^MICU: medical intensive care unit.

^n^CPOE: certified provider order entry.

^o^BP: blood pressure.

Most of the studies included in this review of the literature had financial outcome measures that demonstrated some form of improvement. One of the studies reported that costs that increased during the implementation period were equivalent to the preimplementation level after 6 months [67]. Hepp et al [17] found that the certified physician order entry (CPOE) system (part of the EHR system) generated lower costs in addition to improving medication safety. A few other studies also confirmed that patients in facilities with EHR systems incurred lower costs than those in facilities without an EHR system [54,68,69].

In terms of mixed financial outcomes, the analysis of Adler-Milstein et al [18] exhibited that greater EHR adoption did not improve financial efficiency (measured by the ratio of a hospital’s total expenditures to adjusted patient days) for nonfederal acute care hospitals immediately after the adoption of EHR; however, the results from this study reported improvements in financial efficiency for the years 2010 and 2011 compared with the years 2008 and 2009 [18].

Regarding the reimbursement measure, EHR systems were thought to be responsible for significant improvements in the timeliness of clinical documentation and billing for reimbursement [30,41,76]. The analysis of Cheriff et al [37] documented that physicians who adopted EHRs in a large academic multispecialty physician group captured higher average monthly charges than before the use of EHRs. Similarly, another study reported that the introduction of EHRs was associated with an increase in average per-patient charge and an increase in average per-patient collection [44].

In terms of revenues, profit margins, and return on assets, revenues were reported to have increased in conjunction with EHR adoption [29,51]. A few studies reported improved financial performance concerning savings [42], net profit, and days in account receivables [72] as a result of EHR adoption. One of the studies examined the association among HIT expenses, hospital financial performance, and productivity, with EHR adoption being an intermediate variable. This study indicated a direct and positive association between HIT investment and positive financial performance regarding return on assets [26].

By contrast, a set of results from a survey of ophthalmologists indicated increasing costs and decreasing revenue and productivity with the adoption of EHRs [28]. Other studies have similarly reported findings in terms of a decrease in revenue [54,70] and an increase in cost [29] as a result of EHR adoption. Dandu et al [41] did not provide any statistically significant evidence to report a direct association between EHR adoption and higher-level billing [41]. Similarly, Mirani and Harpalani [27] did not provide any statistically significant evidence to report a direct association between EHR adoption and revenue. Findings from Collum et al [8] suggested that alterations in the level of EHR adoption were not related to increases in revenue and the reduction of operating margins.

### Clinical Outcomes

Of the 58 reviewed studies, 55 (95%) contained segments that were coded under the category of “Value-Clinical Outcomes.” The differing measures for clinical outcomes in these studies were productivity [26,28,30], workflow inefficiency, medical errors, patient safety [3], patient satisfaction, clinical volume, readmission rates, patient LOS [27], and quality indicators at the individual patient level. The different measures of clinical outcomes are listed and described in depth in Table 2. The studies detailed both positive (33/58, 57%), negative (16/58, 28%), and no (7/58, 12%) association relationships between EHR adoption and clinical outcomes. Similar to financial outcomes, an overlap of both positive and negative impacts pertaining to EHR adoption on clinical outcomes was observed in some of the studies.

Most of the clinical outcome measures involved in this review exhibited some form of improvement. The Hessels et al [50] study reported a statistically significant association between EHR adoption and LOS. A significant reduction of LOS in emergency departments [42] and medical errors in emergency and critical care departments [48,49], as well as inpatient acute care settings [62], were indicated as a result of EHR adoption. The rising and falling CPOE rates were also determined to be in correlation with the increase and decrease in LOS [66].

In connection with workflow efficiency and productivity, EHR use was reportedly helpful in improving the promptness of clinical documentation [30], enhancing productivity and efficiency in the workloads of primary care physicians [32], and increasing productivity [37]. Furthermore, EHR was found to be responsible for an increase in patient visits (which results in increased revenue), a decrease in no-show rates (also increasing revenue), and improved care coordination [72]. There was statistically significant progress in terms of completion rates of assessments [29,54], better documentation of medication, patients’ vital signs and pain scores [40], and improved clinical documentation [53,56,76] as a result of EHR adoption.

For the category of patient satisfaction, physicians recognized electronic communication permitted through EHR as a secure and efficient way of communicating with patients, resulting in improvements in patient satisfaction [34]. A study discovered evidence that higher levels of EHR adoption were positively associated with performance and patient satisfaction. This study detected improvements in performance and patient satisfaction for the years 2010 and 2011 compared with the years 2008 and 2009 [18].

With regard to patient safety and medical errors, surgical IT systems (as a subset of EHR systems) positively affected levels of patient safety, compliance, and quality and process measures for patients undergoing surgical procedures in hospitals [31,35]. Outside of surgical IT systems, clinical decision support has also been shown to address other areas of patient safety [59]. For example, adverse drug events decreased by 20% [47], and CPOE was reported to provide exceptional value by improving medication safety in a cost-effective manner [17].

Indicators of quality at the individual patient level, such as rates of antithrombotic therapy and nicotine use documentation, increased immediately following EHR adoption [58]. Similarly, another study reported improvements in antibiotic therapy, blood pressure control, hemoglobin A_1c_ testing, and smoking cessation interventions because of EHR systems [75].

In contrast, for productivity and workload efficiency, the results of a survey indicated that physicians perceived that EHR adoption harmed productivity and increased their workload [28,34,45]. EHR implementation was reportedly associated with increased documentation effort and time, with little to no increase in clinical volume and little to no or perhaps a negative impact on clinical and surgical volume [38,39,41]. Increased documentation time because of EHR adoption resulted in a decrease in the time spent reviewing patient records and performing physical examinations [67]. The results from one of the studies did not identify any differences in productivity (total visit volume) resulting from EHR adoption [70]; however, 3% (2/58) of other studies detailed a decrease in productivity immediately following the adoption of EHR [51,64]. Another example includes significant and consistent decreases in patient volume spanning 4 years after EHR adoption in an academic outpatient ophthalmology practice [55]. EHR systems were said to increase the number of missed assessments, decrease the timely completion rate of assessments, and negatively affect the productivity of clinicians [54]. A study reported that physicians were mostly checking boxes to complete the EHR data process instead of developing or using investigative strategies, which are common among diagnosticians [73].

Considering the patient satisfaction, quality, safety, LOS, and readmission rate perspectives, EHR use resulted in lower patient satisfaction [79] and quality of care [71] for a few years following the adoption of EHRs. In addition, EHR use was associated with an increase in hospital-acquired conditions during EHR implementation [74]. No relationship was found to exist between practice size and the impact of EHR on the quality of patient care from the perspectives of physicians [63]. Some studies reported no association between EHR adoption and improvement in the quality of care provided [36,52,68,78], readmission rates [60], and LOS [48]. Findings from another study that examined physician perceptions of EHRs indicated that physicians believed that EHRs could create new opportunities for error [3].

### The Intersection of Financial and Clinical Outcomes

Having reported on studies that examined financial and clinical outcomes as individual factors, we now report on studies that examined *both* financial and clinical outcomes.

Overall, 9% (5/58) of studies surveyed for this review of the literature reported on the intersection of financial and clinical outcomes. To further investigate this intersection, the category “Value–Intersection of Financial and Clinical Outcomes” was generated. Furthermore, 80% (4/5) of these studies specified a positive association between EHR adoption and financial and clinical outcomes.

In terms of the financial outcomes, hospitals that had adopted EHR selectively increased the efficiency of their turnover rate of Medicare patients to receive higher MU incentives [27]. These findings point toward the impact of EHR adoption on a patient’s stay duration on average (clinical outcome), which, in turn, affects their compensation because of the loss of patient days (financial outcome) from CMS. EHR adoption was associated with enabling the prioritization of improvements in clinical documentation time to improve agency cash flow [30]. EHR use was thought to contribute to shortened emergency department LOS, which led to a positive impact in terms of CMS compensation [42]. Similarly, CPOE, a subset of EHR, was said to be an independent factor in the impact of LOS; therefore, it indirectly contributed to lower costs [66]. By contrast, 20% (1/5) of the studies reported that EHR adoption required a learning period, where increased medical assistant time, patient time, and physician documentation time incurred additional costs [67].

## Discussion

### Principal Findings

The primary goal of this literature review was to substantiate how EHR value is described concerning 2 different outcome categories, financial and clinical outcomes, and to further the exploration of the impact of EHR adoption on these 2 outcome categories. Subsequently, this review incorporated studies that described relationships between EHR adoption along with financial and clinical outcomes with a priori categories (financial outcomes and clinical outcomes) and with an additional category that included the intersection of financial and clinical outcomes. This review of the literature included a total of 58 studies.

Overall, 76% (16/21) of the studies that discussed the financial outcomes of EHR adoption presented a positive relationship between EHR adoption and financial outcomes. These studies observed changes in financial outcomes in terms of profit ratios, costs, revenues, reimbursements, and return on assets. Consistent with the literature, value realization, especially in terms of financial outcomes, is lagging as it involves a large upfront cost [18].

Regarding clinical outcomes, 76% (35/58) of the studies that examined the clinical outcomes of EHR adoption indicated a positive relationship between EHR adoption and clinical outcomes in terms of LOS, readmission rates, patient satisfaction, medical errors, patient safety, user productivity, and quality indicators at individual patient levels. Similar to financial outcomes, value realization regarding clinical outcomes also improved over time. For instance, clinical outcome measures such as rates of hemoglobin A_1c_ testing, recorded BMI, and cholesterol testing decreased before rebounding, following the adoption of EHR [57].

Of the 58 studies in this review of the literature, 5 (9%) studies highlighted the intersection of financial and clinical outcomes. EHR adoption allowed for improvements in clinical documentation time and LOS and sequentially reduced overall costs and improved reimbursement [27,30,42,66]. EHR adoption was also responsible for an increase in personnel costs in association with the new technology’s initial steep learning curve [67]. Overall, these studies indicated interdependence between financial and clinical outcomes, in essence, how one was associated with the other in some form.

This review of the literature discovered some studies with contradictory findings. For example, financial outcomes such as profit margins, return on assets, and costs were some of the measures that reported contradictory findings. A potential reason could be that the studies that reported an inverse relationship reviewed these measures right after the adoption, as opposed to studies that reported it after a longer period. Organizational performance measures such as return on assets, ROI, and return on equity could be examined to explore the cyclical relationship between IT inputs and productivity [80]. Future research may be required to investigate the trajectory and extent of the relationship between IT investments and reinvestments, such as EHR adoption or readoption, and clinical outcomes to further expand upon this question.

### Limitations

The comprehensive findings of this literature review should be considered along with the limitations. Concerning the searched databases, PubMed, Scopus, and Embase—the primary health services and HIT databases—were used. It is possible that studies on the value of EHRs were published outside of health-focused journals and if so, may not have been included in this literature review. Another limitation of this review involves the keywords used in the selection criteria of the article search process. It is possible that the used keywords were not exhaustive, and studies could have been overlooked. Finally, this review included English-only studies that were conducted in the United States. It is possible that other countries with EHRs may have had an experiential understanding that could have contributed to this review. To mitigate bias, manual screening of all the references of included studies was conducted.

### Conclusions

This review of the literature reports on the individual and collective value of EHRs from a financial and clinical outcomes perspective. The collective perspective examined the intersection of financial and clinical outcomes, suggesting a reversal of the current understanding of how IT investments could generate productivity improvements, and prompted a new question to be asked about whether an increase in productivity could potentially lead to more IT investments.

## Abbreviations

CMS: Center for Medicare and Medicaid Services
CPOE: certified physician order entry
EHR: electronic health record
HIT: health IT
HITECH: Health Information Technology for Economic and Clinical Health
LOS: length of stay
MU: meaningful use
PRISMA: Preferred Reporting Items for Systematic Reviews and Meta-Analyses
ROI: return on investment
